# 协同分子印迹技术最新研究亮点

**DOI:** 10.3724/SP.J.1123.2025.12013

**Published:** 2026-02-08

**Authors:** Kaiguang YANG

**Affiliations:** 中国科学院大连化学物理研究所生物技术部，医学蛋白质组学全国实验室，辽宁 大连 116023

分子印迹技术是在模板分子（印迹分子）存在下人工制备分子印迹材料的技术，由于该技术在分子识别领域具有结构预定性、高效识别性和广泛实用性的特点，自现代分子印迹奠基人Guenter Wulff和Klaus Mosbach开创性地提出“共价印迹法”（1972年）^［1］^和“非共价印迹法”（1993年）^［2］^以来，分子印迹技术已经在色谱分离、固相萃取、仿生传感、模拟酶催化、临床药物分析等领域取得了长足的进步。分子印迹材料的核心是识别位点，在共价印迹中，可以选用的可逆共价键有限；在非共价印迹中，常用的非共价键作用力（范德华力、氢键作用力等）存在结合力不足的问题。因此，协同分子印迹技术应运而生，即在印迹位点内，通过多种作用力（如后修饰小分子配基、核酸适配体等）实现对目标分子的高效识别。

近日，中国科学院大连化学物理研究所卿光焱团队在协同分子印迹技术上取得了创新性的进展，针对血液循环系统脓毒症诱发因子脂多糖（LPS）含量低且缺乏特异性抗体的特点，发展了基于结构匹配和特异多肽亲和的协同分子印迹技术，并开发出了高特异性脂多糖吸附材料PS@PA-P_HK_+，为脓毒症精准治疗提供了全新解决方案^［3］^。

在这一技术中，研究团队首先利用噬菌体展示技术，从海量肽库中筛选出一种能够特异性识别LPS家族保守结构核心——Kdo₂-Lipid A的全新肽配体P-HK（序列为：HHHEISWMTWLK）。该肽段对多种细菌来源的LPS均表现出超高亲和力，其结合能力甚至优于传统用于结合LPS的抗生素——多黏菌素B。其次，采用分子印迹技术，构建了具有与LPS几何匹配空腔的聚合物颗粒（PS@PA+）。为克服LPS因结构多变、两亲性及相容性差导致的印迹难题，研究团队开创了一种新型乳液界面聚合法，即在水包油的溶液体系内加入LPS分子，具有亲水糖链和疏水脂链的LPS会以表面活性剂的方式定向排列在液滴表面。随着聚合物微球的形成与模板LPS的洗脱，微球表面形成了LPS分子的印迹空腔，且其疏水部分的印迹孔可与Lipid A精确匹配，进一步保证了材料与多数细菌LPS吸附的普适性。该分子印迹以苯乙烯和二乙烯苯作为疏水骨架，亲水功能层由聚（乙二醇）甲基醚丙烯酸酯（PEGMEA）与丙烯酸（AA）构成，其中PEGMEA具有抗蛋白黏附功能，AA能够为亲和肽提供链接位点，向聚合体系内引入LPS模板分子，并利用其在油水界面定向组装的特性，确保了在整个聚合过程中空腔的精准形成，成功制备了具有几何匹配空腔的分子印迹聚合物PS@PA+。最终，通过将高亲和力的P-HK肽共价接枝到印迹聚合物表面，得到了新型吸附材料PS@PA-P_HK_+（图1）。该材料通过亲和肽P-HK的高亲和性结合与分子印迹孔穴的空间构型匹配，二者产生协同增效作用，从而实现了对血液中痕量LPS的高效与特异性捕获。

**图1:**
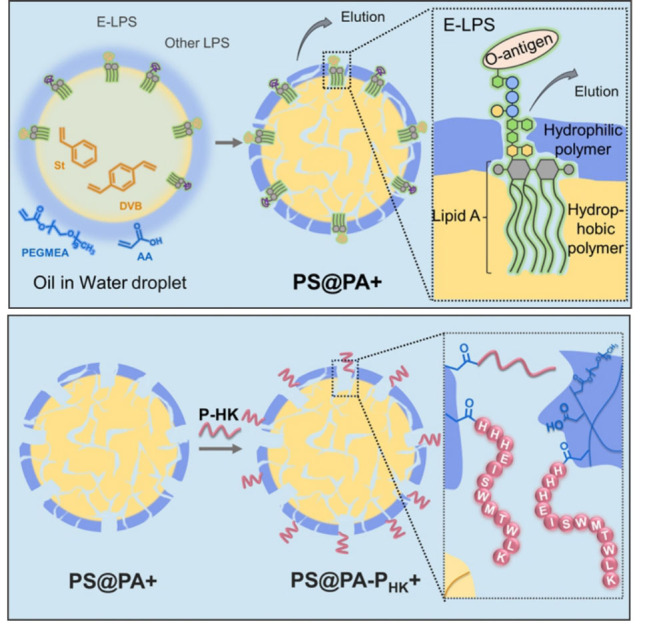
基于结构匹配和特异多肽亲和的协同分子印迹技术

该材料对大肠杆菌的LPS的清除率高达99.2%，吸附容量达到543 EU/mg，即便在成分复杂的全血中，其清除率仍保持在95.4%，显示出强大的吸附和抗干扰能力。此外，该材料具备“广谱”清除潜力。它对鼠伤寒沙门氏菌、鲍曼不动杆菌等多种病原菌的LPS也表现出高达82.6%~88.5%的清除率，意味着未来有望用于应对不同病原体引发的复杂感染。在生物安全性方面，该材料表现优异：不会引起溶血，对凝血功能无显著影响，细胞毒性测试也证实其生物相容性良好，为后续临床转化奠定了安全基础。在脓毒症兔模型的治疗实验中，结果更令人振奋：使用该材料进行血液灌流治疗后，血液中循环LPS水平显著下降84.8%；关键炎症因子白细胞介素-6（IL-6）浓度大幅降低，有效遏制了“炎症风暴”；病理分析显示，治疗能显著减轻肺、肝、肾等多器官损伤，且治疗组兔子在24 h后的生存率达到100%，而对照组生存率仅为40%。充分证明了协同分子印迹技术在脓毒症治疗中的强大潜力。

该协同分子印迹技术的优越性在于：（1）由于LPS的O抗原多糖部分结构多变，且内毒素分子本身具有两亲性的特点，当前大多数非共价分子印迹的聚合物体系难以适用，而乳液界面聚合法保证了LPS模板与聚合物产生稳定的非共价键力且能在油水界面定向组装，确保了在聚合过程中空腔的精准形成，成功制得了具有几何匹配空腔的分子印迹材料。（2）在精准几何匹配空腔的基础上，该研究创新性地将靶向多肽与空间位点进行复合，显著提高了LPS的清除效率。（3）该协同印迹技术具有普适性，该策略可进一步拓展至其他血液中毒素（如过量细胞因子、胆红素、肌酐等）的精准清除，为尿毒症、自身免疫性疾病、重症感染等疾病的精准血液净化技术发展开辟了新的道路。
